# Competitive environments sustain costly altruism with negligible assortment of interactions

**DOI:** 10.1038/srep02836

**Published:** 2013-10-03

**Authors:** C. Patrick Doncaster, Adam Jackson, Richard A. Watson

**Affiliations:** 1 Centre for Biological Sciences, Institute for Life Sciences, University of Southampton, Southampton SO17 1BJ, UK; 2 Natural Systems Group, Electronics & Computer Science, Institute for Life Sciences, University of Southampton, Southampton, SO17 1BJ, UK

## Abstract

Competition hinders the evolution of altruism amongst kin when beneficiaries gain at the expense of competing relatives. Altruism is consequently deemed to require stronger kin selection, or trait-selected synergies, or elastic population regulation, to counter this effect. Here we contest the view that competition puts any such demands on altruism. In ecologically realistic scenarios, competition influences both altruism and defection. We show how environments that pit defectors against each other allow strong altruism to evolve even in populations with negligible kin structure and no synergies. Competition amongst defectors presents relative advantages to altruism in the simplest games between altruists and defectors, and the most generic models of altruistic phenotypes or genotypes invading non-altruistic populations under inelastic density regulation. Given the widespread inevitability of competition, selection will often favour altruism because its alternatives provide lower fitness. Strong competition amongst defectors nevertheless undermines altruism, by facilitating invasion of unrelated beneficiaries as parasites.

An act of strong altruism involves giving a fitness advantage to others at net personal cost to the benefactor^1^,^2^. Strong altruism presents a special case in evolutionary biology because its cost to the altruist appears to contradict the self-interested incentives of natural selection. Its resilience to defection despite the cost is explained by the indirect benefits that return to an altruist from interactions that are positively assorted by kin recognition, population viscosity, reciprocity, or other structuring mechanisms^3^,^4^,^5^. The evolution of strong altruism is inhibited, however, when the very structures that promote indirect benefits also promote competition amongst the beneficiaries^6^. This benefits-cancelling effect of competition may sustain only weak altruism, without net cost to the altruist, or suppress cooperation altogether^7^. A challenge remains therefore to explain the widespread occurrence of altruistic traits, and cooperative behaviours generally, in crowded environments^8^,^9^,^10^,^11^,^12^.

Strong altruism occupies the +/− quartile of pairwise interaction space in the Hamiltonian classification^3^,^13^ completed by +/+ mutually beneficial (including weakly altruistic), −/+ selfish, and −/− spiteful interactions. The +/− interaction of strong altruism, henceforth referred to as ‘altruism', can achieve an evolutionarily stable strategy (ESS) when its beneficiaries are kin. Specifically, kin selection must satisfy Hamilton's rule: 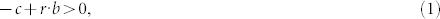
in which the altruist's net cost *c* to personal fitness in delivering fitness benefit *b* is compensated by beneficiaries with coefficient of relatedness *r* returning inclusive fitness *r*·*b* to the altruist^3^. Coefficient *r* quantifies the benefits arising from positively assorted interactions. In pairwise interactions, assortative mixing is a necessary pre-requisite for altruism by kin selection, and/or trait selection on synergistic benefits^4^,^14^.

Altruism is inhibited when relatives compete for its benefits^6^,^8^,^15^,^16^. Competition means that one individual's gain is another's loss; competitive interactions amongst relatives therefore result in beneficiaries gaining from altruism only at the expense of other relatives of the altruist. For example, if mutual altruism raises personal fitness in the form of *b* − *c* extra offspring, then their displacement of other relatives of the altruist by population regulation incurs an inclusive fitness cost *r*′·(*b* − *c*), where *r*′ is the relatedness of those relatives to the altruist. Hamilton's rule can accommodate this supplementary cost by reconfiguring relatedness with respect to the ‘economic neighbourhood' that encompasses competition with relatives, with a devaluation of *r* that has the effect of inhibiting altruism^15^. Unless *r*′ < *r*, altruism is unsustainable with inelastic population regulation^8^,^16^. Recent life-cycle models have shown how elasticity in regulating mechanisms can offset this inhibitory effect of competition, even to the extent that competition favours altruism when it brings trait-selected synergistic benefits^11^,^12^,^17^.

Even with elastic population regulation and synergistic benefits, the requirement for strong population structure and/or positive synergies to overcome inhibition by competition assumes that the driver for altruism is an opportunity to collect the positive benefits of the altruistic act. In ecologically realistic scenarios of crowding, however, the driver for altruism can be something altogether more mundane and ubiquitous: release from competition amongst non-altruists. Competition is widespread and largely inevitable in the natural world, and conditions that provide positive synergies are possibly too rare to explain the ubiquity of altruism. Here we contest the prevailing view that altruism requires stronger kin or trait selection in crowded conditions, by showing that its models have yet to embrace fully the evolutionary tenet that traits spread when their carriers have higher fitness than the population average (even if the trait carries no intrinsic benefit). Prior work on the evolution of altruism has focused solely on the impacts of altruists on beneficiaries, calibrating Hamilton's rule against a non-altruism alternative of no interaction. Whilst this alternative is appropriate to density-independent dynamics, it ignores a basic principle of population regulation, that the payoff for mutual competition is negative relative to no interaction.

We consider an environment in which competition lowers the payoff for non-altruists with other non-altruists, as well as for altruists with beneficiaries. For example cooperative hunting and breeding in groups of African wild dogs (*Lycaon pictus*) brings fitness benefits that may depend on prevailing conditions of competitor density^18^. The alternative to altruism amongst kin in this globally competitive environment is competition amongst non-altruist (‘defector') kin. Their competition with each other presents a bleak prospect against which altruism prevails relatively easily, even with negligible population structure, and without requirement for *r*′ < *r*, or elastic regulation, or synergistic benefits. We find that competitive environments facilitate altruism by devaluing its alternatives, as opposed to improving its opportunity. This prediction is consistent with the observation in wild dogs that group size increases individual fitness more strongly under higher competitor density^18^. We demonstrate the broad scope of our theoretical analysis by modelling it with the simplest games between altruist and defector strategies, and the most generic dynamics of altruistic phenotypes and genotypes invading a density-regulated population of non-altruists. These games and models underpin understanding of all empirical cases of cooperative behaviours amongst taxa ranging from bacteria to vertebrates, and we point to examples of both conferred benefits and public goods benefits. We discuss the reasons why density regulation amongst non-altruists has been ignored in previous theory of altruism in the presence of competition.

We start with a conventional Prisoner's Dilemma game, in which unilateral defection pays better than mutual altruism and mutual defection pays better than unilateral altruism. We interpret its pairwise interactions as products of density-dependent competition, and we analyse the inclusive fitness required to escape the dilemma. We then model the influence of interactions amongst defectors on the threshold of relatedness necessary for an ESS with altruism, always assuming a Prisoner's Dilemma for personal fitness. Our method aligns with recent life-cycle models in recognizing that competition and inclusive fitness have independent causal factors, of resource limitation and population structure respectively^11^,^12^. Accordingly, we decouple the effects of competition from those of inclusive fitness by allocating all competitive effects to payoffs in personal fitness. Inclusive fitness is then calculated on payoffs resulting from interactions that include competition, instead of being calculated as a supplement to pre-competition inclusive fitness. This approach greatly simplifies accounting procedures by obviating the need to specially add the effects of competition into the inclusive fitness payoffs. Thus we treat the change in personal fitness *b* − *c* for mutual altruism as the average payoff obtained in the presence of competition amongst altruists selected with average relatedness *r*. Competition may render *b* − *c* negative relative to no interaction, and we consider both positive and negative scenarios. Model predictions depart from those of previous theory only when we factor in the presence of competition to the payoff for mutual defection. Competition renders this payoff negative relative to no interaction, and we therefore refer to it as a fitness cost *d* (see ref. 19 for discussion of such costs generally). We will demonstrate that this method of accounting for competition perfectly maps the game-theoretic payoffs onto Lotka-Volterra interaction coefficients for density-dependent population dynamics.

## Results

### Altruism in a competitive environment

**Figure 1:**
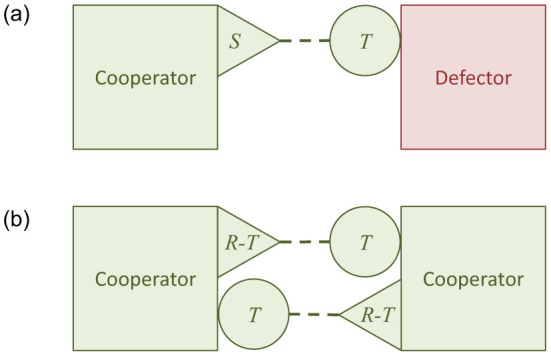
Hamilton's inclusive-fitness model applied to pairwise interactions. (a) Cooperator-Defector and (b) Cooperator-Cooperator, following the schema of ref. 25. Given players of equal fitness before interacting (squares), each interaction (dashed connector) has payoffs for actor (triangle) and recipient (circle) that take Table 1a parameters for *b* as a conferred benefit, and Table 1b parameters for *b* as a public good. The inclusive fitness payoff for each player is the summed effect of its actions on itself plus the effect of its actions on others weighted by relatedness *r*^24^.

Altruists escape the Prisoner's Dilemma by kin selection returning an inclusive fitness payoff *S^i^* to the altruist interacting with a defector, which exceeds the payoff *P^i^* to a defector with another defector. Table 1 shows how the conventional application of Hamilton's rule with *P* = *P^i^* = 0 requires: *S* + *r*·*b* > 0, where payoff *S* accounts for the net personal cost to the altruist in accordance with condition (1). The value of *S* depends on whether the altruism involves conferring a benefit at net cost *c* (Table 1a) or manufacturing a public good at net cost *c* − *b* (Table 1b). Figure 1 illustrates Hamilton's model schematically for both these types of altruism in the two-player case.

**Figure 2:**
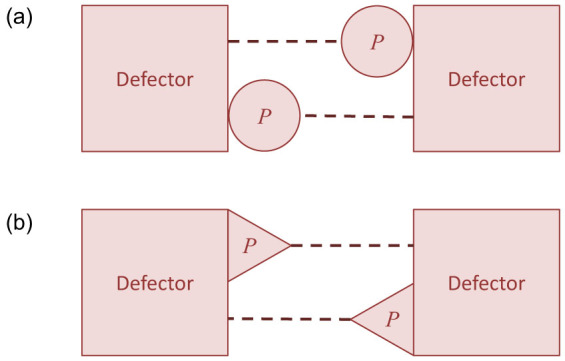
Alternative types of Defector-on-Defector interaction with payoff *P* = −*d*. Schema as for Fig. 1. (a) Mutual displacement (i.e., each intruding on the other, for example animals intruding into each other's territories or plants encroaching on each other's canopies or at the population level any rise in mortality resulting from resource competition), setting inclusive fitness *P^i^* = *r*·*P*. (b) Mutual retreat (i.e., each withdrawing away from the other, for example animals avoiding conflict or at the population level any reduction in fecundity by an animal or plant resulting from resource limitation), setting *P^i^* = *P*.

We now build ecological realism into the game by giving the interaction between non-altruists a personal cost *d*, setting *P* = −*d*. The condition *S^i^* > *P^i^* for an ESS with altruism then requires a generalized form of Hamilton's rule: 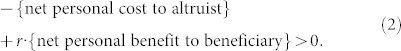
The presence of personal cost *d* lowers the threshold of condition (2), thereby favouring persistence of altruism. Depending on the type of interaction expressed by *d*, its associated inclusive fitness payoff *P^i^* either increments the inclusive fitness benefit to each altruist or it decrements their net personal cost. Figure 2 illustrates schematically the derivations of *P^i^* given *P* = −*d* that define alternative formulations of Hamilton's rule. If *d* accounts for a competitive impact of actor on recipient, such as displacement, then *P^i^* = *r*·*P* (Fig. 2a). Consequently, the condition *S^i^* > *P^i^* for an ESS with altruism in the presence of mutual displacement amongst non-altruists requires 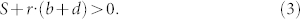
In effect, competition cost *d* supplements the net personal benefit of altruism to the beneficiary that contributes to inclusive fitness of the altruist. Alternatively, if *d* accounts for a competitive impact on the actor, such as retreat from conflict, then *P^i^* = *P* (Fig. 2b). Consequently, *S^i^* > *P^i^* given mutual retreat amongst non-altruists requires 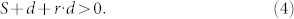
In effect, competition cost *d* subtracts from the net personal cost of altruism. Whichever the circumstance, costly altruism is more easily favoured when set against costly interactions amongst non-altruists.

### Invasion of altruism into a population of non-altruists

Where payoffs have an ESS outcome in relative frequencies of two strategies (as opposed to a Nash equilibrium in relative probability of each strategy for two players), Lotka-Volterra population dynamics set criteria for ESS outcomes that are identical to those of two players, as described in the Methods. A more costly interaction amongst Defectors, and therefore more negative payoff *P* = −*d*, facilitates invasion by a Cooperator phenotype or genotype into a density-regulated population. Exactly as for two-player games, the condition *S^i^* > *P^i^* for escaping a Prisoner's Dilemma has alternative constructions depending on the derivation of *d*. Condition (3) applies if *d* accounts for displacement; condition (4) applies if *d* accounts for retreat. At the population level, the displacement effect of *d* may be expressed by raised adult mortality and retreat by inhibited recruitment (fecundity or juvenile survival).

Beyond the particular context of the Prisoner's Dilemma game, the same predictions arise in a standard derivation of Hamilton's rule from a positive selection gradient. A population is susceptible to invasion by altruism given a positive difference between the fitness of carriers of the altruism allele and the fitness of those carrying an alternative defector allele: *W*(altruist) − *W*(defector) > 0. For example, if competition expresses retreat, and *w*_0_ is the unavailable intrinsic fitness before any density-dependent interactions, the fitness equations are: 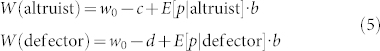
where *p* is the relative frequency of cooperators amongst interactions with the focal individual. The invasion condition is therefore: −*c* + *d* + *r*·*b* > 0, which is condition (4) above with explicitly defined *r* = *E*[*p*|altruist] − *E*[*p*|defector]. A value of *d* = 0 returns Hamilton's rule in the conventional form of condition (1). A value of *d* > 0 simply recognizes that density-dependent competition does not allow individuals to avoid interacting with each other, meaning that *W*(defector) = *w*_0_ is not an option in the absence of altruism. Likewise for competition expressed by displacement, in the light of Fig. 2 the fitness equations are: 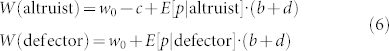
and the invasion condition is therefore: −*c* + *r*·(*b* + *d*) > 0, which is condition (3) above.

### Altruism with negligible assortment of interactions

For two-player probabilities and for population-level frequencies alike, a mixed ESS is possible even with *r* = 0 provided only that *S* > *P* (i.e., *d* > *c* for a conferred benefit, or *d* > *c* − *b* for a public good). The +/− interaction thereby sustained without assortative mixing cannot be classified as altruism because it cannot be driven by the benefactor. Rather, it is a parasite-host interaction driven by the beneficiary acting as a parasite (as described in the Methods). What distinguishes an altruist from a victim-host is its +/− interaction being sustainable only in the presence of assortative mixing (i.e., *S^i^* > *P^i^* despite *P* ≥ *S*). Accordingly, conventional formulations of Hamilton's rule with *d* = 0 find that altruism with higher cost relative to benefit requires stronger kin selection.

**Figure 3:**
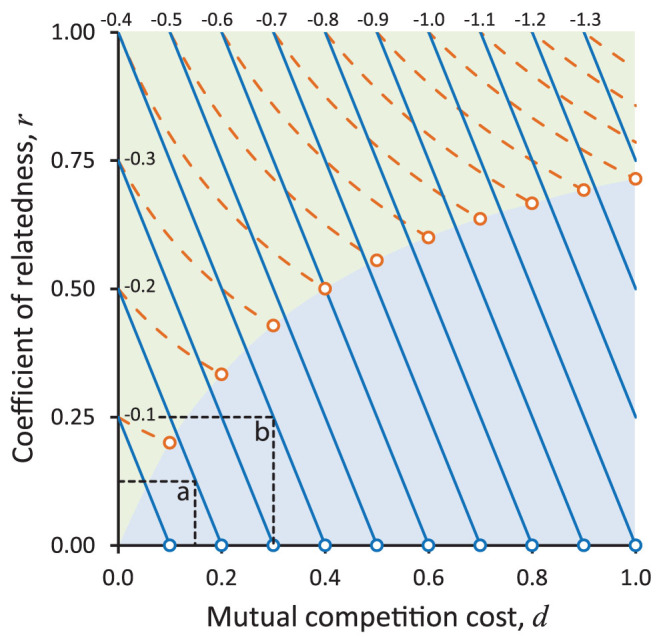
Threshold relatedness *r* above which the ESS supports altruism. An altruist incurs a payoff *S* < 0 to itself in giving benefit *b* = 0.4 to another, here showing alternative *S* = −0.1, −0.2 … −1.3 (lines left to right in each colour). For any given *S*, the graph shows that the threshold *r* declines with higher competition cost *d* of mutual defection, whether competition expresses displacement (dashed orange lines, condition (3)) or retreat (solid blue lines, condition (4)); open circles mark the point on each line beyond which altruism gives way to parasitism, by virtue of *S* > *P*. Green region sustains altruism with displacement or retreat competition, blue region sustains altruism with retreat only. (a) Retreat *d* = 0.15 sustains acts of altruism with *S* = −0.2 (i.e., net personal cost of *c* = 0.2 for a conferred benefit, or *c* = 0.6 for a public good) amongst relatives with *r* > 0.125; (b) retreat *d* = 0.3 sustains altruism with *S* = −0.4 (i.e., conferred *c* = 0.4, or public good *c* = 0.8) amongst relatives with *r* > 0.25.

Altruism has no such dependency on *r*, however, in the presence of a cost *d* for mutual defection. Figure 3 illustrates the thresholds of conditions (3) and (4) (dashed and solid lines respectively) to show how *d* > 0 sustains higher-cost and lower-benefit altruism, and with a declining threshold of *r* for larger *d*. Token levels of assortative mixing sustain even the most costly altruism, given sufficiently large cost *d* in competition by mutual retreat amongst non-altruists. Consider an example of public-good manufacture with *b* = 0.4 and *c* = 0.6 (which sets *S* = −0.2). In the absence of competition, the altruist achieves an ESS only if kin selection exceeds full-sib relatedness (*r* > 0.5). In the presence of Defector-on-Defector competition, however, Fig. 3a shows that kin selection need only exceed full cousin relatedness (*r* > 0.125) to sustain altruism if retreat *d* = 0.15, and it needs only token relatedness if retreat *d* = 0.2. Consider an alternative example of a conferred benefit *b*. In the absence of competition, no amount of relatedness can sustain it when the altruist incurs cost *c* = *b*. In the presence of Defector-on-Defector competition, however, Fig. 3b shows that kin selection need only exceed half-sib relatedness (*r* > 0.25) to sustain altruism when *b* and *c* = 0.4 (so *S* = −0.4) if retreat *d* = 0.3, and it needs only token relatedness if retreat *d* = 0.4.

Strong competition amongst Defectors nevertheless threatens altruism with invasion of beneficiaries as parasites of benefactors. The +/− interaction takes on the character of a parasite-host relationship upon meeting the condition *S* > *P*. The Fig. 3 open circles mark the cusp of an altruist's beneficiary functioning as a parasite. For competition expressed by mutual retreat, the condition is not met until *r* = 0 meaning that this kind of competition benefits altruism at all levels of relatedness. Competition by mutual displacement, however, benefits altruism only at higher levels of relatedness, before the altruism gives way to parasitism. Appendix S1 in the Supporting Information contains a graphical spread-sheet calculator for enumerating ESS outcomes of conditions (3) and (4) for any specified fitness payoffs and *r*.

## Discussion

For two players and for population dynamics of two phenotypes and two genotypes alike, a Cooperator that persists by virtue of personal fitness *S* > *P* will function as a host to a parasitic Defector genotype that sustains the ESS without assortative mixing^19^. It belongs to the +/− parasite-host quartile of pairwise interaction space in the Lidickerian classification^20^ completed by +/+ mutualistic, −/+ host-parasite, and −/− competitor interactions. Here we have extended game theory to address the domain between conventional altruism and parasitism, respectively bounded in Fig. 3 by *d* = 0 in the Hamiltonian classification and by *r* = 0 in the Lidickerian classification. The unifying framework has allowed us to re-evaluate the conditions in which altruism can invade a competitive environment of non-altruists. Our findings give a new perspective to the well-established and tested prediction that competition amongst altruists inhibits the evolution of altruism^6^,^8^,^15^,^16^. The competitive impact that is conventionally modelled as suppressing the inclusive fitness benefits of altruism is easily offset by competition amongst non-altruists diminishing the benefits of defection. Strong altruism consequently requires little population structure in the presence of competition. All of social evolution theory is underpinned by the principle that population structure explains altruism, and our study downgrades its influence.

We have defined the benefit *b* and cost *c* of strong altruism as being the positive and negative payoffs observed in the presence of competition. In principle, it might seem desirable to exclude from *c* any fraction that is imposed by competition, in order to count only the amount owing to adaptations that evolved for the purpose of providing benefit to others^2^. A model for altruism in competitive environments could be constructed this way, for example by subtracting a constant *d* from all interactions in the Table 1 payoff matrix. This would change the interpretation quantitatively, but not qualitatively. It would explain the persistence of high-cost altruism with little assortment of interactions in terms of a cost that mostly derives from competitive impact. The small portion of the benefactor's cost of interaction that is not due to competition (the altruistic part) would then be sustained according to Hamilton's rule, in the conventional form of condition (1) rather than the expanded form of condition (2).

We have chosen not to construct our models in this way, because to do so presupposes that it is possible to separate the cost of altruism from the cost of competition. We doubt that they can be separated either in principle or in practice. Precisely how a negative payoff to an altruist partitions into components due to altruism only and competition only is a matter determined by evolutionary history that cannot be assessed merely by establishing that the altruist now incurs a negative payoff. Moreover, the presence of altruism and the presence of competition may now be interdependent, making it impossible to separate them experimentally. We find it more informative to interpret *b* and *c* as benefits and costs in the presence of competition, because these are the directly observable fitness consequences of the interaction between benefactor and beneficiary. Furthermore, the direct translation of this interpretation into Lotka-Volterra dynamics (detailed in the Methods) facilitates the distinction of altruism from parasitism and other forms of competition^19^. With this broader definition of the net personal benefit and cost of an altruistic interaction, a strongly negative payoff *S* is explained in terms of high-cost altruism being more easily favoured when set against costly interactions amongst non-altruists. Ref. 21 describes an empirical example consistent with such an interpretation, in which competition within and between termite species promotes cooperation by exacerbating the impact of internal conflicts.

We suggest that the altruism we see in nature may often result, in large part, from the average fitness of non-altruists being diminished by competition, and in that case, the conditions necessary to sustain it are broad and do not require strong kin selection. We suspect that many natural environments present variable conditions for altruism, depending on current resource availability and threats to the integrity of altruism from parasitism. An altruistic net-cost transferral of fitness can degenerate into a parasitic transferral of identical magnitude (i.e., same *S* and *T*) solely upon a reduction in *P* sufficient to cross the threshold *S* > *P*. Empirical studies of cooperation face a further substantial challenge in reliably distinguishing strong altruism, motivated by the benefactor and sustained by indirect benefits, from parasitism, motivated and sustained by the beneficiary^19^.

Life-cycle models of synergistic altruism have recently shown how competition favours altruistic traits that create population elasticity, for example by increasing growth yield^11^, or carrying capacity^17^. The presence of costs to defecting from altruism, as modelled here, will augment any synergies that contribute to raising the relative payoffs for altruism. Given the evident prevalence of such costs of defection under density regulation, their influence on the threshold of relatedness that sustains altruism begs an empirical evaluation. We are not aware of explicit tests to date, and we thus conclude with a disjunction. If conditions more commonly present altruists that are advantaged by synergies, then conventional analyses depending on highly assorted interactions will have most relevance. However, if conditions more commonly present non-altruists as being disadvantaged by competition, the new analysis shows that high assortment will have less relevance for sustaining altruism.

The decoupling of competition from inclusive fitness has allowed a novel mapping of Hamilton's rule with Lotka-Volterra population dynamics, within the unifying framework of game theory for pairwise interactions. A correct interpretation requires acknowledging that cooperative interactions such as altruism which are normally associated with social games can apply also to conflict games for unsocial settings such as density-dependent competition. The simple switch from *P* ≥ 0 for social games to *P* < 0 for conflict games opens game-theoretic applications of evolutionary biology to the full spectrum of possibilities for +/− interactions. These range from Hamiltonian altruism without competition through to high-cost and low-benefit altruism enabled by negligible assortment of competitors, and ultimately into victims of parasitism.

## Methods

### Prisoner's Dilemma and related two-strategy games

The Prisoner's Dilemma encapsulates the problem that altruism is not a stable outcome amongst freely mixing interactions. Consider a Cooperator strategy that confers fitness benefit *b* on another at net cost *c* to the benefactor, and its interaction with a non-reciprocating Defector strategy. The Cooperator's altruism might take the form of helping another to raise offspring at a cost to its own reproduction. The Prisoner's Dilemma game allocates per capita fitness payoffs for unilateral (cross-strategy) interactions as ‘Sucker' *S* = −*c* to a Cooperator with a Defector, and ‘Temptation' *T* = *b* to a Defector with a Cooperator, and per capita fitness payoffs for mutual interactions as ‘Reward' *R* = −*c* + *b* to a Cooperator with a Cooperator, and ‘Penalty' *P* = 0 to a Defector with a Defector^22^. Unilateral defection then pays better than mutual cooperation (*T* > *R*), and mutual defection pays better than unilateral cooperation (*P* > *S*). Regardless of starting strategies, the stable outcome is therefore mutual defection, despite its lower payoff than that for mutual cooperation (*R* > *P*).

Amongst all possible two-strategy games, the Prisoner's Dilemma belongs to the class of games with a pure ESS outcome of Defectors only, which is set by *T* > *R* and *P* ≥ *S*^19^,^23^. The Prisoner's Dilemma is opposed by the Harmony Game, which belongs to the class with a pure ESS outcome of Cooperators only, set by *R* ≥ *T* and *S* > *P*. Between these two extremes, a Stag-Hunt game belongs to the class with bi-stability, which is set by *R* ≥ *T* and *P* ≥ *S*. Alternatively, the Hawk-Dove or Snowdrift game belongs to the class with a mixed ESS outcome of Cooperators and Defectors, which is set by *T* > *R* and *S* > *P*. The equilibrium probability of being a Cooperator is then (*S* − *P*)/(*S* − *P* + *T* − *R*), and the alternative probability of being a Defector is (*T* − *R*)/(*S* − *P* + *T* − *R*)^23^.

### Apportioning inclusive fitness in two-strategy two-player games

An altruist escapes the Prisoner's Dilemma when positively assorted interactions give it indirect fitness benefits sufficient to cancel its direct net cost *c*. The positive assortment is quantified by the coefficient of relatedness *r*, which measures the component of relatedness resulting from assortative mixing. This is given by the average covariance in the identities of the interacting pair relative to the average covariance without assortment^24^,^25^.

Table 1 shows how relatedness can change the game outcome from an ESS of pure defection to an ESS with cooperation. This happens when the inclusive fitness payoffs break at least one of the conditions of the Prisoner's Dilemma by achieving *S^i^* > *P^i^*, despite having personal fitness payoffs *P* ≥ *S*. In Table 1a with *P* = *P^i^* = 0, an inclusive fitness benefit *r*·*b* that more than cancels a net personal fitness cost *c* changes the game, in accordance with Hamilton's rule (condition (1)). The Prisoner's Dilemma in personal payoffs then becomes a Harmony Game with a pure-strategy ESS of mutual cooperation, by virtue of inclusive fitness meeting both defining conditions of this alternative game: *S^i^* > *P^i^* and *R^i^* ≥ *T^i^*.

Table 1b shows the criteria applied to manufacture of a public good, which benefits both parties, as opposed to conferral of a benefit only on the recipient. For example, some bacterial cells manufacture polymers for the production of an extracellular matrix of ‘biofilm', which others may also use as a refuge^22^. Again, players escape the Prisoner's Dilemma on Hamilton's rule for the inclusive fitness benefit *r*·*b* more than cancelling the net personal fitness cost *c* − *b* of its manufacture.

### Phenotypic altruist invading a density-regulated population

The rate of change over continuous time in relative frequencies *x*_C_ and *x*_D_ of Cooperator and Defector phenotypes in a large population has 1-dimensional Lotka-Volterra dynamics: 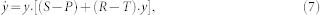
where *y* = *x*_C_/*x*_D_^19^,^26^. A Cooperator strategy invades the population on condition *S* > *P*, whereupon its ratio with a Defector strategy grows logistically to equilibrium y* = (*S* − *P*)/(*T* − *R*) on condition *T* > *R*, or it excludes the Defector if *R* ≥ *T*. These conditions for Cooperator invasion align precisely with those of two players for the Prisoner's Dilemma (no invasion), Stag-Hunt (bi-stability), Harmony Game (displacement of Defectors), and Hawk-Dove or Snowdrift game (coexistence).

With payoffs *S*, *T* and *R* set by *c* and *b* according to Table 1, Cooperator invasion is facilitated by smaller *P*, due for example to competition amongst Defectors. If *P* < 0, Cooperators that meet the invasion criterion in terms of personal fitness *S* > *P*, will be hosts to Defector parasites that sustain the ESS without assortative mixing, in a Hawk-Dove contest or Snowdrift game^19^,^23^. Although a host has benefit extracted from it by others, it reduces the cost to itself if it can limit the interaction to kin, for example by limiting dispersal (a form of ‘social niche construction'^27^,^28^). A host that manages in this way to recoup some indirect benefit to itself acts as an ‘incidental altruist', with its altruism being incidental to the +/− interaction enforced by parasitism^19^. Our focus in this analysis is on the alternative scenario, of a +/− interaction that is sustained by altruism (i.e., not by parasitism). Cooperators will equilibrate to an ESS that is sustained by altruism if they meet the invasion condition only in terms of inclusive fitness *S^i^* > *P^i^* (while *P* ≥ *S*). We analyse this condition in the Results section.

The population dynamics of equation (7) accommodate the known inhibitory effect of competition between altruists^6^,^8^,^15^. A low or negative value of *R* expresses the inhibition, just as a negative value of *P* expresses competition between non-altruists. A lower value of *R* brings a lower equilibrium frequency of altruists (if *P* ≥ *S*), or hosts to parasitism (if *S* > *P*). The competition between altruists may be offset, however, by synergies in mutual cooperation^11^,^14^. Synergies may increase growth yield, for example when wolves reduce their individual hunting costs by pack-hunting prey that are larger than the sum of individually hunted prey. Synergies can also raise carrying capacity, for example when bacteria manufacture polymers for biofilm production. These examples count as synergies if the mutual helping raises *R* above its Table 1 value as set by *c* and *b* in the presence of competition. The synergy can be enumerated as a supplement *a* to the *R* payoff in Table 1, for the added value of the synergy to personal fitness. Mutual interactions then involve strong altruism only if *c* > *a*; otherwise with *a* ≥ *c* the game has a pure ESS of Cooperators with mutual interactions that incur no net cost to the actor.

### Genotypic altruist invading a density-regulated population

The same boundary conditions apply also to two competing populations of independently self-replicating genotypes. These might be two strains of bacteria, for example, only one of which manufactures polymers for the formation of a biofilm that provides refuge to both^22^. The rate of change over continuous time in *n_i_* individuals of population *i* has 2-dimensional Lotka-Volterra dynamics: 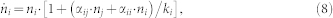
where *α_ij_* is the per capita impact on genotype *i* from genotype *j*, relative to *i* on itself: *α_ii_* = −1; *k_i_* is the carrying capacity of genotype *i* in the absence of the other genotype; and the genotype-*i* specific timescale is normalized against its per capita rate of increase before any competition^19^,^26^.

This conventional Lotka-Volterra model for competing populations translates directly to game-theoretic terms for Cooperator and Defector genotypes as: 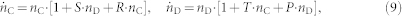
where *S* = *α*_CD_/*k*_C_, *R* = *α*_CC_/*k*_C_, *T* = *α*_DC_/*k*_D_, *P* = *α*_DD_/*k*_D_. At the stable equilibrium *n*_C_* and *n*_D_* of equations (9), *n*_C_* > 0 requires *S* > *P*, and *n*_D_* > 0 requires *T* > *R*, just as for phenotype invasions (though achieved by 2-dimensional instead of 1-dimensional dynamics^19^). Note that the normalized coefficients *α*_CC_ and *α*_DD_ for population regulating competition within each genotype force *R* and *P* negative. A Cooperator genotype with +/− interaction set by *T* > 0 > *S* may yet invade a Defector population, with the invasion facilitated by *k*_C_ > *k*_D_ which sets *R* > *P*. In effect, the Cooperator's intrinsic efficiency in resource utilization offsets its costly interaction with the Defector genotype.

Cooperators achieve an ESS with altruism when they meet the invasion condition only in terms of inclusive fitness: *S^i^* > *P^i^* (i.e., while *P* ≥ *S*). The coefficient of relatedness *r* that makes this possible quantifies the assortment of interactions with a Defector genotype that brings indirect benefits to an altruistic Cooperator's offspring^29^.

## Author Contributions

Study ideas by C.P.D., R.A.W. and A.J. Study design and analysis by C.P.D., who wrote the paper with contributions from R.A.W. and A.J. All authors contributed substantially to revisions of earlier drafts.

## Supplementary Material

Supplementary Information Supplementary Info 1

## Figures and Tables

**Table 1:** Matrix of payoffs for two-strategy games with +/− unilateral payoffs (*T* > 0 > *S*)

	Cooperator	Defector
(**a**) Conferral of benefit *b* at net cost *c*		
Cooperator	*R* = −*c* + *b*,	*S* = −*c*,
	*R^i^* = *R* − *T* + *r*·*b*	*S^i^* = *S* + *r*·*b*
Defector	*T* = *b*,	*P*,
	*T^i^* = 0	*P^i^*
(**b**) Manufacture of public good *b* at net cost *c* − *b* > 0		
Cooperator	*R* = −*c*/2 + *b*,	*S* = –(*c* – *b*),
	*R^i^* = *R* – *T* + *r*·*b*	*S^i^* = *S* + *r*·*b*
Defector	*T* = *b*,	*P*,
	*T^i^* = 0	*P^i^*

Each cell shows the payoff to the row strategy for its interaction with the column strategy, expressed as personal fitness (*R*, *S*, *T*, *P*), and as inclusive fitness (*R^i^*, *S^i^*, *T^i^*, *P^i^*) given average relatedness *r*.

## Notes

Appendix S1. Graphical spread-sheet calculator for enumerating ESS outcomes of conditions (3) and (4) for any specified *r* and fitness payoffs in two-strategy pairwise interactions. Requires Microsoft Excel.
